# Comment on “Serum Hepcidin and Soluble Transferrin Receptor in the Assessment of Iron Metabolism in Children on a Vegetarian Diet”

**DOI:** 10.1007/s12011-018-1241-1

**Published:** 2018-01-10

**Authors:** Piotr Rzymski, Tomas Ganz

**Affiliations:** 1 Department of Environmental Medicine, Poznan University of Medical Sciences, Poznań, Poland; 2 Departments of Medicine and Pathology, David Geffen School of Medicine, University of California, Los Angeles, USA

Dear Editor,

We read with great interest the paper by Ambroszkiewicz et al. [1] entitled “Serum hepcidin and soluble transferrin receptor in the assessment of iron metabolism in children on a vegetarian diet” that was published in the journal of Biological Trace Element Research (DOI: 10.1007/s12011-017-1003-5). It presents the results of an observational study of markers of iron status in children aged 4.5–9.0 years on lacto-ovo-vegetarian (*n* = 43) or omnivorous diet (*n* = 46). The research compared biochemical parameters (serum concentration of ferritin, transferrin, soluble transferrin receptor, hepcidin, iron, hemoglobin, and C-reactive protein) and erythrocyte parameters, as well as estimated energy and nutrient intakes, between the two groups. The vegetarian children had a two-fold decrease in serum hepcidin level accompanied by decreased ferritin level and slight but statistically significant increase in concentration of soluble transferrin receptor (sTfR), but no differences in concentration of hemoglobin, mean corpuscular volume, iron, and transferrin compared to the omnivorous group. Moreover, vegetarian children had comparable total iron intake but consumed more (approx. by 30%) ascorbic acid in food [1]. The paper suggests that subclinical iron deficiency in vegetarian children is manifested by elevated sTfR concentration and decreased hepcidin. Here, we would like to present an alternative view that these changes may reflect a clinically benign form of adaptation for more efficient iron absorption and utilization.

Owing to the increasing popularity of vegetarian diets, their clinical consequences are becoming clearer and include such potential health benefits as decreased all-cause mortality and decreased risks of obesity, type 2 diabetes, and coronary heart disease [2]. Reputable medical societies, including Academy of Nutrition and Dietetics (USA), acknowledge that appropriately planned vegetarian diets, including the vegan form, are suitable for all stages of the life cycle, including pregnancy, lactation, infancy, childhood, adolescence, and older adulthood, and even for athletes [3]. However, unbalanced vegetarianism can be as harmful as an unbalanced omnivorous diet [4].

Most data on the health effects of vegetarian diets were collected from adults, so the study by Ambroszkiewicz et al. [1], despite its small sample size, is an important contribution to the field. The diet of children is largely determined by their caregivers and it is understandable that some (including caregivers already practicing some form of vegetarianism) will fear that restriction of meat consumption may lead to deficiencies and adversely affect the development of their children. This is particularly important in the case of iron as it plays a central role in many key biological processes, including intermediary metabolism, energy production, and oxygen delivery to tissues [5]. Globally, iron deficiency is recognized as a major health problem known to be associated with serious neurodevelopmental and cognitive deficits in low-resource settings [6]. However, it is still not clear to what extent these serious problems depend on coexisting nutritional deficiencies, alcohol and drug use during pregnancy, endemic infections, and other confounding factors that are uncommon among persons in high-resource settings who adopt vegetarianism as a lifestyle choice. In this regard, it is somewhat reassuring that even very severe isolated iron deficiency, with anemia and severe microcytosis caused by mutations in TMPRSS6 (matriptase 2), did not cause developmental deficits in children diagnosed in high-resource settings [7, 8].

The study by Ambroszkiewicz et al. [1] reported no significant difference in iron intake between the two groups. Some authors argue that, in vegetarians, iron intake needs to be much higher (up to 80%) to overcome the lower biological availability of iron forms in their diet [9]. We question this recommendation because it does not sufficiently consider factors that enhance iron absorption in vegetarians. Higher intake of ascorbic acid in vegetarians supports the reduction of the trivalent iron to its more soluble and absorbable divalent form [10]. Moreover, iron absorption is increased in subjects with low serum hepcidin or its surrogate, low serum ferritin [11]. The study by Ambroszkiewicz et al. [1] observed higher intake of ascorbic acid in vegetarian children and acknowledged that this may partially counteract the iron-sequestering effects of polyphenols and phytic acid in a vegetarian diet [12]. Furthermore, habitual consumption of high-phytate foods may reduce the negative effect of phytate on non-heme iron absorption [13].

Ambroszkiewicz et al. [1] noted the increased concentrations of sTfR (mean 1.33 vs 1.12 mg/L, *p* < 0.01) and decreased hepcidin level (5.46 vs 11.54 ng/L; *p* < 0.05) in lacto-ovo-vegetarian children. Based on these findings, the authors suggest that the vegetarian children may suffer from subclinical iron deficiency. Their view is supported by two references in which similar trends in hepcidin and sTfR levels were found in children diagnosed with iron deficiency [14, 15]. However, as yet, no reference ranges of sTfR and hepcidin levels have been established for children. Moreover, in Ambroszkiewicz et al. [1], the mean difference in sTfR concentration between lacto-ovo-vegetarian and omnivorous children was only 18% (0.21 mg/L). Although serum ferritin was lower in lacto-ovo-vegetarians than that in omnivores, it is notable that, in every case, it fell within the established reference range of ≥ 12 μg/L in cases of children aged below 5 years and ≥ 15 μg/L for children older than 5 years of age [16]. Using the recently proposed criteria to detect clinically significant iron deficiency in children by decreased mean corpuscular volume of erythrocytes [17], only four children in each group could be considered to have borderline microcytosis.

We would like to suggest that instead of diagnosing a potential disease condition in the lacto-ovo-vegetarian children, the biochemical changes could be considered a form of adaptation wherein a slight increase in sTfR and two-fold decrease in hepcidin as observed by Ambroszkiewicz et al. [1] are evidence of homeostatic changes that increase iron utilization and absorption from diets that contain less bioavailable iron. Increased sTfR reflects higher expression of TfR in erythrocyte precursors, allowing more efficient iron uptake even when plasma iron concentrations are decreased. There is also evidence that increased TfR expression helps mediate suppression of hepcidin in the liver [18]. By degrading duodenal ferroportin, hepcidin is a key regulator of iron uptake from the diet [19, 20]; therefore, its lower circulating concentrations should enhance iron absorption from diets with restricted bioavailable iron. Moreover, vegetarian children demonstrated a significantly increased intake of vitamin C [1] which may promote iron absorption. It would be very interesting to follow up on children studied by Ambroszkiewicz et al. [1] within the next 2–4 years to ascertain whether any of them will have developed clinical iron deficiency.

Finally, simple measures can be taken to enhance the availability of dietary iron in children or adults who do develop clinical iron deficiency on vegetarian diets. The consumption of diets containing ferritin-rich seeds (legumes, nuts, corn) provides iron in a more bioavailable form than other vegetables [21]. In some countries, certain foods (e.g., cereals) are fortified with iron and represent an acceptable dietary supplement for many vegetarians.

In summary, there is no evidence that the adaptive changes described by Ambroszkiewicz et al. [1] in vegetarian children in high-resource settings indicate any adverse effects on their well-being or development. We recognize that such children have not been adequately studied and encourage further research in this area.
